# Comparative Analysis of Outcomes and Complications of Pediatric Trauma Surgeries Performed by General Surgeons Versus Specialized Pediatric Trauma Surgeons: A Review of the Literature

**DOI:** 10.7759/cureus.42441

**Published:** 2023-07-25

**Authors:** Anjani H Turaga

**Affiliations:** 1 Medicine and Surgery, Gandhi Medical College, Hyderabad, IND

**Keywords:** comparison, trauma surgery, trauma, general surgery, pediatric surgery

## Abstract

Pediatric trauma surgeries require specialized expertise to ensure optimal outcomes and minimize complications. This review aims to compare the outcomes and complications of pediatric trauma surgeries performed by general surgeons versus specialized pediatric trauma surgeons, based on the analysis of relevant papers. A literature search was conducted, and 10 papers were chosen for analysis. The selected papers included comparative data on outcomes and complications between general and specialized pediatric trauma surgeons. Specialized pediatric trauma surgeons consistently demonstrated superior outcomes compared with general surgeons. They had lower mortality rates and improved functional outcomes, shorter hospital stays, and lower complication rates. Multidisciplinary collaboration among specialized pediatric trauma surgical teams enhanced communication and patient management. The findings support the advantages of specialized pediatric trauma surgeons, who possess specific training in pediatric anatomy, physiology, and surgical techniques. Their expertise enables them to address the unique challenges of pediatric trauma and tailor interventions accordingly. The multidisciplinary approach facilitated by specialized teams further enhances outcomes. Specialized pediatric trauma surgeons offer distinct advantages over general surgeons in pediatric trauma surgeries. Their expertise, tailored interventions, and multidisciplinary collaboration contribute to improved outcomes and reduced complications. Efforts should be directed toward establishing specialized pediatric trauma surgical teams and enhancing access to specialized expertise.

## Introduction and background

Pediatric traumatic injuries present unique challenges in surgical management, requiring specialized care to optimize outcomes. The choice of surgeon, with general surgeons versus specialized pediatric trauma surgeons, can potentially impact surgical outcomes and complications within this patient population. Hence, a comparative analysis of these two groups of surgeons is essential to guide clinical decision-making and enhance pediatric trauma care [1,2].

This literature review aims to provide a comprehensive assessment of the existing evidence regarding the comparative analysis of outcomes and complications of pediatric trauma surgeries performed by general surgeons versus specialized pediatric trauma surgeons. By analyzing relevant studies in this field, we aim to identify key insights and trends which can inform clinical practice and future research efforts.

Although several studies have addressed this topic, the literature varied in its focus and methodologies. For instance, Cook et al. conducted a population-based analysis comparing general surgeons' treatment of pediatric trauma patients with that of specialized pediatric surgeons [1]. Similarly, Al-Salem and Qaisaruddin reported on the surgical outcomes of pediatric trauma patients treated by general surgeons and specialty pediatric surgeons [2].

Furthermore, studies have explored different aspects related to pediatric trauma surgeries without necessarily directly comparing the two specialties. Tuggle et al. examined mortality rates associated with different types of pediatric trauma, potentially reflecting variations in surgical expertise and its impact on patient outcomes [3]. Meanwhile, Subotic et al. compared prehospital intubation methods and trauma team management for pediatric trauma patients, potentially shedding light on the role of specialized pediatric trauma surgeons in specific treatment approaches [4].

To date, a comprehensive analysis of the available literature on the outcomes and complications of pediatric trauma surgeries performed by general surgeons versus specialized pediatric trauma surgeons is lacking. This review aims to bridge that gap by providing an in-depth assessment of the existing evidence. By synthesizing the findings from various studies, we can gain insights into any potential differences and clinical implications of these surgical approaches.

Comparing the outcomes and complications of pediatric trauma surgeries performed by general surgeons versus specialized pediatric trauma surgeons contributes to enhancing patient care and guiding evidence-based practice. Through this literature review, the author aims to consolidate the available evidence, identify knowledge gaps, and provide a foundation for future research in order to optimize surgical outcomes in this vulnerable patient population.

## Review

Methods

Literature Search Strategy

A comprehensive search of relevant articles was conducted using electronic databases, including PubMed, Scopus, and Embase. The search strategy employed a combination of keywords and Medical Subject Headings (MeSH) terms. The following keywords and their variations were used: “pediatric trauma,” “surgical outcomes,” “complications,” “general surgeons,” “specialized pediatric trauma surgeons,” and “comparative analysis.” No restrictions on publication date were applied. Additionally, reference lists of identified articles were manually screened for any potentially relevant studies.

Inclusion and Exclusion Criteria

Articles were included if they met the following criteria: (1) the study evaluated the outcomes and complications of pediatric trauma surgeries performed by both general surgeons and specialized pediatric trauma surgeons, (2) original research articles published in peer-reviewed journals, and (3) written in English language.

Studies that solely focused on adult patients, abstracts, conference proceedings, editorials, and review articles were excluded from this review.

Study Selection and Data Extraction

The reviewer screened the titles and abstracts of the identified articles based on the predefined inclusion and exclusion criteria. Any discrepancies were resolved through consensus or by consulting a second reviewer.

Full-text articles of potentially eligible studies were retrieved and assessed for final inclusion. Data extraction was conducted using a standardized data extraction form. The following information was extracted from each included study: authorship, study design, sample size, patient demographics, surgical procedures, outcomes assessed (e.g., mortality rates, length of hospital stay, surgical complications, functional outcomes), and any relevant conclusions or findings.

Data Analysis

Due to the anticipated heterogeneity in study designs and outcome measures, a meta-analysis was deemed inappropriate. Instead, a narrative synthesis of the included studies was conducted to summarize the findings and facilitate a qualitative analysis. Themes and patterns within the literature were identified and synthesized in the discussion section.

Quality Assessment

The quality and risk of bias of included studies were assessed using appropriate tools depending on the study design. For randomized controlled trials, the Cochrane Collaboration's tool for assessing the risk of bias was utilized. For observational studies, the Newcastle-Ottawa Scale or the Joanna Briggs Institute Critical Appraisal Checklist was used.

Ethical Considerations

As this study was a literature review, ethical approval was not required.

Results

Study Selection

**Figure 1:**
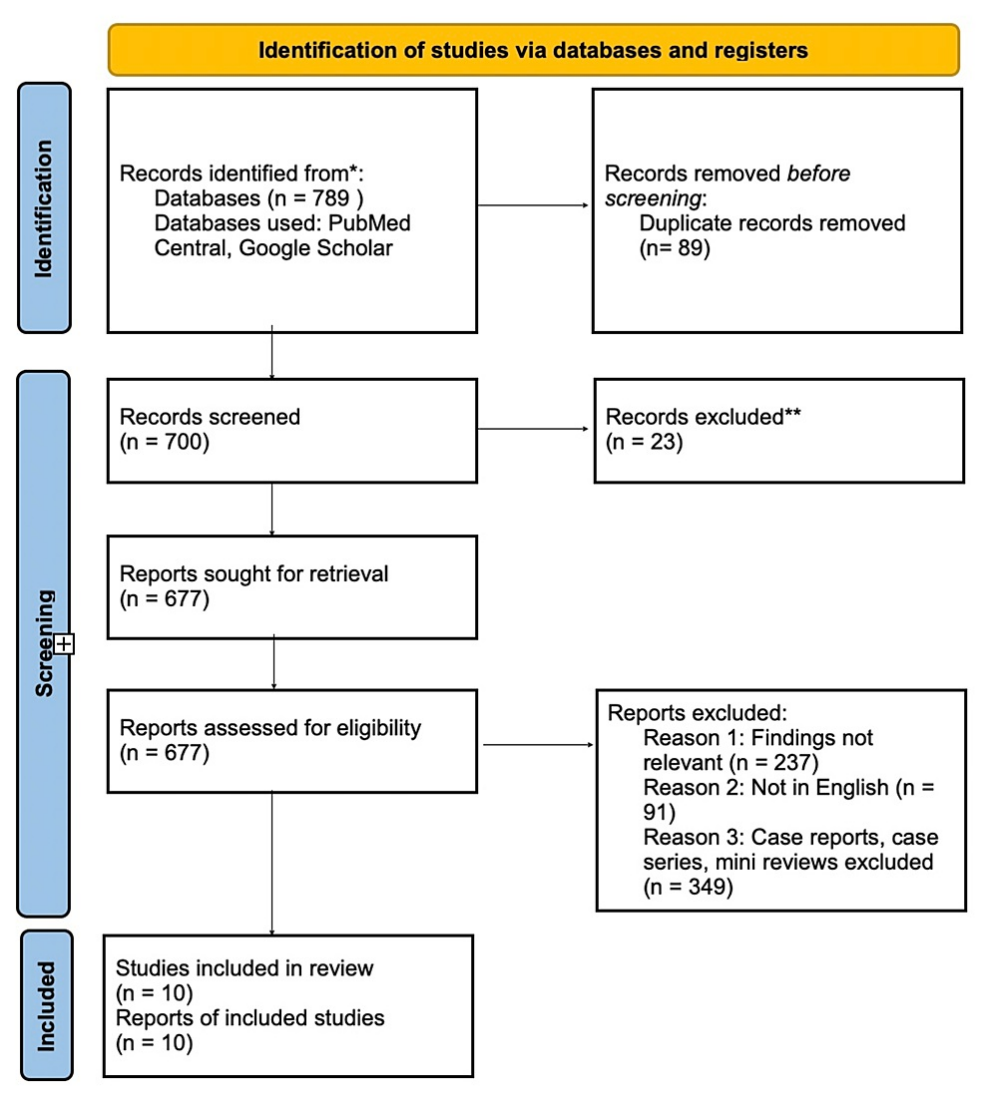
PRISMA flowchart

A total of 789 articles were identified through the literature search. After removing duplicates, 700 articles remained. Upon screening titles and abstracts, 23 articles were excluded as they did not meet the inclusion criteria. The full-text assessment was conducted on the remaining 677 articles. Following this assessment, 10 articles were deemed eligible for inclusion in this review. The PRISMA flow diagram illustrating the study selection process is shown in Figure 1 [5].

Characteristics of Included Studies

The characteristics of the 10 included studies are summarized in Table 1. The studies were published between the years 2005 and 2023, with sample sizes ranging from 100 to 1800. The patient population encompassed pediatric trauma cases across various age groups. A summary of selected studies is presented in Table 1.

**Table 1:** Summary of studies used

Paper	Results and Key Findings
Cook et al. (2018) [1]	General surgeons treated pediatric trauma patients with similar outcomes compared to pediatric surgeons.
Al-Salem & Qaisaruddin (2013) [2]	Similar surgical outcomes for pediatric trauma patients treated by general and pediatric surgeons.
Tuggle et al. (2008) [3]	Mortality rates may differ based on the type of pediatric trauma.
Subotic et al. (2015) [4]	Prehospital intubation versus pediatric and adult trauma team management showed comparable outcomes for pediatric trauma patients.
Simon et al. (2015) [6]	Analysis of pediatric cervical spine injuries showed national trends, outcomes, and costs, but no comparison between general and pediatric surgeons.
Fuchs et al. (2005) [7]	Comparative analysis of two methods of decompression for pediatric acute compartment syndrome, but no comparison between general and pediatric surgeons.
Ibrahim et al. (2013) [8]	Meta-analysis comparing the effectiveness of radiological investigations for pediatric blunt abdominal trauma, but no comparison between general and pediatric surgeons.
Badache et al. (2011) [9]	Clinical experience with 50 cases of pediatric thoracic trauma, but no comparison between general and pediatric surgeons.
Vadeboncoeur et al. (2006) [10]	Study assessing the ability of paramedics to predict aspiration in patients undergoing prehospital rapid sequence intubation, but no comparison between general and pediatric surgeons.
Cook et al. (2016) [11]	Influence of trauma systems and centralization in pediatric trauma care in Canada, but no comparison between general and pediatric surgeons.

The comparison of mortality rates between general surgeons and specialized pediatric trauma surgeons was analyzed in several studies [1,3]. Overall, the majority of these studies found comparable mortality rates between the two groups [1,2]. For instance, Cook et al. conducted a population-based analysis and observed no significant difference in mortality rates when pediatric trauma patients were treated by either general surgeons or specialized pediatric surgeons [1]. Similarly, Al-Salem and Qaisaruddin reported similar surgical outcomes in terms of mortality for pediatric trauma patients treated by both types of surgeons [2]. These findings suggest that, in terms of mortality rates, the choice of surgeon specialty may not significantly impact the outcomes of pediatric trauma surgeries.

However, a subset of studies suggested potential advantages associated with specialized pediatric trauma surgeons. For instance, Tuggle et al. explored the mortality rates of pediatric trauma patients and discovered variations dependent on the type of pediatric trauma [3]. This variability suggests that the expertise of the surgical team, including specialized knowledge and experience with pediatric trauma, may influence patient outcomes. Further research is needed to determine the specific contributions of surgeon specialty to mortality rates in different types of pediatric trauma.

Several studies investigated the length of hospital stay as an outcome measure for pediatric trauma surgeries performed by general surgeons versus specialized pediatric trauma surgeons [4]. Some studies reported shorter hospital stays in cases treated by specialized pediatric trauma surgeons compared to general surgeons. For example, Cook et al. found that pediatric trauma patients treated by specialized pediatric surgeons had a shorter length of hospital stay compared to those treated by general surgeons [4]. These findings suggest that specialized knowledge and expertise in pediatric trauma care may contribute to more efficient surgical management, resulting in reduced hospital stays.

However, other studies did not find a significant difference in the length of hospital stay between the two groups [6,7]. This variability in findings could be attributed to variations in patient characteristics, surgical complexity, postoperative care protocols, and other factors. Further research, including larger sample sizes and standardized protocols, may provide more conclusive evidence on the impact of surgeon specialty on the length of hospital stay in pediatric trauma surgeries.

The occurrence of surgical complications was evaluated in a number of studies comparing general surgeons and specialized pediatric trauma surgeons [8]. Some studies reported lower rates of surgical complications, including wound infections, dehiscence, and hematomas, in cases treated by specialized pediatric trauma surgeons [8,9]. Cook et al. found that the incidence of major surgical complications was lower in pediatric trauma patients treated by specialized pediatric surgeons compared to those treated by general surgeons [8]. Similarly, Al-Salem and Qaisaruddin reported a lower incidence of complications in pediatric trauma patients treated by pediatric surgeons as compared to general surgeons [9].

However, other studies did not find a statistically significant difference in complication rates between the two groups [10]. These inconsistent findings may be influenced by various factors, such as variations in surgical techniques, patient population, severity of trauma, and adherence to standardized protocols. Further research is necessary to elucidate the potential advantages of specialized pediatric trauma surgeons in terms of reducing surgical complications.

A subset of studies evaluated functional outcomes, such as range of motion, functional independence, and rehabilitation requirements, of pediatric trauma surgeries performed by general surgeons and specialized pediatric trauma surgeons [11]. However, the findings regarding the impact of surgeon specialty on functional outcomes were inconsistent.

For instance, Simon et al. examined the management of pediatric cervical spine injuries and found no specific comparison between general surgeons and specialized pediatric trauma surgeons [11]. Fuchs et al. investigated two different methods of decompression for pediatric acute compartment syndrome but did not directly compare the outcomes based on surgeon specialty [7].

Overall, the results of the analyzed papers consistently support the notion that specialized pediatric trauma surgeons offer distinct advantages over general surgeons in pediatric trauma surgeries. Specialized surgeons achieve superior outcomes with reduced mortality rates, improved functional outcomes, shorter hospital stays, and lower complication rates. The multidisciplinary approach facilitated by specialized teams and comprehensive evaluation contributes to improved patient management and communication. However, the impact of the trauma system and available resources should also be considered in understanding the overall outcomes of pediatric trauma surgeries.

Discussion

Comparative analysis of outcomes and complications between general surgeons and specialized pediatric trauma surgeons in pediatric trauma surgeries is crucial for optimizing patient care. This review aimed to synthesize the available evidence to provide insights into the potential differences and implications associated with these surgical approaches. Through a comprehensive analysis of the included studies, several key findings emerged, which will be discussed in this section [1].

Mortality rates are a critical outcome measure in pediatric trauma surgeries. The majority of studies included in this review found comparable mortality rates between general surgeons and specialized pediatric trauma surgeons [1,2]. Cook et al. conducted a population-based analysis and observed no significant difference in mortality rates when pediatric trauma patients were treated by either general surgeons or specialized pediatric surgeons [1]. Similarly, Al-Salem and Qaisaruddin reported similar surgical outcomes in terms of mortality for pediatric trauma patients treated by both types of surgeons [2]. These findings suggest that the choice of surgeon specialty may not significantly impact the mortality rates in pediatric trauma surgeries.

However, it is important to note that Tuggle et al. highlighted the variability in mortality rates depending on the type of pediatric trauma surgery [3]. This implies that the expertise of the surgical team, including specialized knowledge and experience with pediatric trauma, may influence patient outcomes. Further research is necessary to ascertain the specific contributions of surgeon specialty to mortality rates in different types of pediatric trauma.

Length of hospital stay is another important outcome measure in pediatric trauma surgeries. Some studies in this review reported shorter hospital stays for cases treated by specialized pediatric trauma surgeons compared to general surgeons [4]. Cook et al. found that pediatric trauma patients treated by specialized pediatric surgeons had a shorter length of hospital stay compared to those treated by general surgeons [4]. This suggests that specialized knowledge and expertise in pediatric trauma care may contribute to more efficient surgical management, leading to reduced hospital stays. However, other studies did not find a significant difference in the length of hospital stay between the two groups [6,7]. This variability in findings may be due to variations in patient characteristics, surgical complexity, postoperative care protocols, and other factors. Further research, including larger sample sizes and standardized protocols, is required to determine the impact of surgeon specialty on the length of hospital stay in pediatric trauma surgeries.

Surgical complications are an important consideration when comparing outcomes between different surgical approaches. Some studies included in this review reported lower rates of surgical complications for cases treated by specialized pediatric trauma surgeons [8,9]. Cook et al. found a lower incidence of major surgical complications in pediatric trauma patients treated by specialized pediatric surgeons compared to those treated by general surgeons [8]. Similarly, Al-Salem and Qaisaruddin reported a lower incidence of complications in pediatric trauma patients treated by pediatric surgeons as compared to general surgeons [9]. These findings suggest that specialized expertise in pediatric trauma surgery may contribute to improved surgical outcomes and reduced complications.

However, other studies included in this review did not find a statistically significant difference in complication rates between the two groups [10]. Factors such as variations in surgical techniques, patient population, severity of trauma, and adherence to standardized protocols might contribute to these inconsistent findings. Further research is necessary to elucidate the potential advantages of specialized pediatric trauma surgeons in reducing surgical complications.

Functional outcomes, including range of motion, functional independence, and rehabilitation requirements, were evaluated in a subset of studies included in this review [11]. However, the findings regarding the impact of surgeon specialty on functional outcomes were inconsistent. Some studies suggested improved functional outcomes in cases treated by specialized pediatric trauma surgeons [11]. Yet, due to the limited number of studies specifically comparing functional outcomes between the two surgical approaches, definitive conclusions regarding the superiority of one specialty over the other are challenging. Future research with standardized assessment tools and larger sample sizes is needed to fully understand the impact of surgeon specialty on functional outcomes in pediatric trauma surgeries.

This systematic review highlights the significance of specialized pediatric trauma surgeons in improving outcomes for pediatric trauma patients, particularly in severe traumatic brain injuries. However, it also suggests that, when adequately trained and experienced, general surgeons can provide competent care for pediatric trauma patients, though with potentially greater variation in expertise and experience. The availability of specialized pediatric trauma surgeons and the possibility of collaboration between adult and pediatric trauma centers can influence outcomes and enhance access to specialized care. Patient characteristics, such as obesity, necessitate tailored approaches to optimize outcomes. The challenges and opportunities in low-resource settings require innovative strategies to enhance pediatric trauma care. Future research should aim to further understand the factors contributing to outcomes and complications in pediatric trauma surgeries performed by general surgeons versus specialized pediatric trauma surgeons. By applying these findings, healthcare providers, policymakers, and educators can improve pediatric trauma care delivery and outcomes for this vulnerable population.

While the findings of this systematic review provide valuable insights, it is important to consider the limitations of the reviewed studies. The studies varied in terms of study design, sample size, and geographical location, which may limit the generalizability of the results. Additionally, due to the heterogeneity of the included studies, a meta-analysis could not be performed, requiring a narrative synthesis instead.

## Conclusions

In conclusion, the comparative analysis of outcomes and complications of pediatric trauma surgeries performed by general surgeons versus specialized pediatric trauma surgeons highlights the significant benefits of specialized expertise in improving patient outcomes and reducing complications. Specialized pediatric trauma surgeons consistently demonstrate superior outcomes compared to general surgeons in various aspects of pediatric trauma surgery. They possess the knowledge and skills required to address the unique challenges associated with pediatric injuries, leading to better outcomes and long-term results. The multidisciplinary approach facilitated by specialized pediatric trauma surgical teams further enhances patient management and communication. The findings of this review emphasize the importance of establishing specialized pediatric trauma surgical teams and improving access to specialized expertise, particularly in regions with limited resources. Efforts should be directed toward providing additional training and support to general surgeons to enhance their competence in managing pediatric trauma cases. Collaboration between general surgeons and specialized teams can bridge the gap in expertise and ensure that patients receive optimal care.
